# How Heuristic Credibility Cues Shape Perceived Credibility on Social Media: A Meta-Analysis of Experimental Research

**DOI:** 10.3390/bs16071184

**Published:** 2026-07-13

**Authors:** Renjun Cao, Norliana Binti Hashim, Saiful Nujaimi Abdul Rahman

**Affiliations:** Faculty of Modern Languages and Communication, Universiti Putra Malaysia, Serdang 43000, Malaysia; h_norliana@upm.edu.my (N.B.H.); nujaimi@upm.edu.my (S.N.A.R.)

**Keywords:** social media, heuristic credibility cues, perceived credibility, source cues, social cues, meta-analysis

## Abstract

As the information environment evolves, social media has become the primary channel through which the public accesses and shares information, and perceived credibility has emerged as a critical influence on how users evaluate the credibility of information. Existing research suggests that heuristic credibility cues can enhance users’ perceived credibility, yet the findings remain inconsistent. Consequently, it is necessary for researchers to systematically examine whether heuristic credibility cues can effectively enhance perceived credibility. This study employed a meta-analysis to analyse 18 studies meeting the selection criteria, involving a total sample size of 14,188 participants. The aim was to assess the overall effect of social media heuristic credibility cues on perceived credibility and to explore the influence of potential moderating mechanisms on perceived credibility. The results indicate that manipulating source cues and social cues, which serve as heuristic credibility cues on social media, significantly increased perceived credibility (g = 0.307, *p* < 0.001). Effect sizes varied across moderating variables such as the type of heuristic credibility cue, participant type, method of measuring perceived credibility, experimental design, sample size, and year of publication. Among these, the type of heuristic cue and participant type were significant moderators; specifically, authoritative sources were more effective than other types of information sources in enhancing perceived credibility; the impact of different types of social cues on perceived credibility was also significant to varying degrees. Furthermore, student groups were more susceptible to the influence of heuristic credibility cues than non-student groups. These findings provide theoretical and practical insights for the design of information dissemination and the construction of perceived credibility on social media. It should be noted that, given the limited number of studies included in this meta-analysis and the restricted range of moderator variables, the above conclusions require further empirical research to be tested and confirmed.

## 1. Introduction

Perceived credibility on social media refers to an individual’s overall cognitive assessment of the credibility, accuracy and reliability of the information they encounter, based on their own subjective judgement. This concept is rooted in the classic source credibility framework proposed by Hovland and Weiss (1951), which was subsequently extended to the digital media environment by Flanagin and Metzger (2000). Since the 1950s, researchers in psychology and communication studies have been dedicated to defining and measuring source credibility. Early research by Hovland and his colleagues demonstrated that while highly credible sources are more persuasive, information itself is acquired equally from both highly credible and low-credibility sources. Tseng and Fogg (1999) indicate that credibility is not an inherent property of the information itself, credibility is not an inherent property of the information itself, but rather a subjective assessment formed by the recipient of the information regarding its credibility, reliability and acceptability in a specific circumstance; essentially, it is a perception that exists within an individual’s cognitive judgement. Consequently, perceived credibility reflects a broader subjective assessment rather than a direct equivalent of trust.

In the social media environment, perceived credibility is crucial to user behaviour, such as engagement. Because social media platforms rely on mobile and internet technologies, enabling users to conveniently access, create, edit, share and interact. However, users do not always regard information from social media as reliable information. Compared to traditional media, the public’s perceived credibility remains relatively low (Newman et al., 2023), reflecting widespread concerns in the digital environment regarding misinformation and the credibility of social media (Lazer et al., 2018). Low levels of perceived credibility pose risks to the legitimacy and sustainability of social media. Flanagin and Metzger (2000) argue that, unlike users in traditional media who act as information consumers, social media users may simultaneously assume multiple roles like content creators, disseminators and consumers. Faced with a vast amount of information, perceived credibility has become increasingly important in the evaluation process and is regarded as a key indicator for predicting users’ attitudes and behavioural intentions.

However, perceived credibility evaluation on social media is considerably more complex than in the traditional media environment. This stems from several factors. Firstly, the significant increase in content creators on social media means that users may not only receive information from the authorities but also frequently come into contact with content created by opinion leaders, ordinary users and influencers simultaneously; AI and algorithmic recommendations play a significant role in this processing. Secondly, information spreads rapidly in diverse forms, and during this process, systematic and comprehensive screening and verification are often lacking. Finally, when accessing information on social media, users may confuse the source of the information with the content itself; this situation complicates the assessment of perceived credibility. In addition, the absence of uniform standards for information quality, the susceptibility of information to manipulation and tampering, the lack of clear contextual cues, and the coexistence and mutual influence of multiple credibility assessment targets (such as content, sources and platforms) are also contributing to the relative complexity of evaluating perceived credibility. Against this backdrop, users often have no choice but to rely on their own subjective judgement to form a subjective perception of whether information is credible, reliable or trustworthy, that is perceived credibility (Flanagin & Metzger, 2000; Mitra et al., 2017).

To make judgements in information-rich and uncertain environments, users always use heuristic credibility cues to assess credibility (Hilligoss & Rieh, 2008). Drawing on heuristic processing theory and research on online credibility, heuristic credibility cues refer to informational or contextual signals that enable users to rapidly assess information credibility while operating under limited cognitive effort (Sundar, 2008). Previous research indicates that perceived credibility assessments tend to rely less on systematic verification and are instead more heavily influenced by heuristic cues such as source, social endorsement, platform availability, visual presentation, and emotional framing (Metzger et al., 2010), although different types of heuristic credibility cues vary in their specific forms and underlying psychological mechanisms, they share a common theoretical function in the credibility judgement process as informative or contextual cues that reduce cognitive cost, thereby helping users to rapidly form credibility assessments in information-rich and uncertain environments. Drawing on the theory of heuristic information processing, this study does not integrate these cues based on consistency in their form or mechanisms of action, instead treating them as heuristic judgement cues that facilitate the formation of credibility assessments on the basis of their shared theoretical attributes, and incorporating different types of heuristic credibility cues into a comprehensive analysis, among these cues, source cues and social cues have become the most influential core cues in social media credibility research, and continue to be tested.

Source cues refer to heuristic signals related to the information provider’s professional competence, authority, trustworthiness, or institutional legitimacy. Existing research consistently indicates that, in digital media environments, source credibility has been identified as one of the most robust predictors of perceived credibility. Flanagin and Metzger (2007) found that source reputation and perceived authority are the primary heuristic cues used by users when assessing credibility online. Bauer and Clemm von Hohenberg (2021) further noted that perceived news credibility is significantly shaped by both the authenticity of the content and source credibility, while collective social endorsement signals may play a relatively limited role. Similarly, Morris et al. (2012) investigated the relationship between verified Twitter accounts and users’ perceived credibility, finding that verified accounts are one of the most classic source cues and have a significant impact on users’ perceived credibility. Dobber et al. (2023) used traffic-light veracity labels to examine the impact of such labels on source credibility and message credibility, and similarly found that heuristic trust cues have a marked effect on enhancing users’ perceived credibility. Prike et al. (2024) directly manipulated credibility badges and found that such badges can enhance the ability to distinguish genuine information and reduce the spread of misinformation. Javed et al. (2024) and Vu et al. (2025) indicate that users’ perceptions of information credibility depend primarily on the professionalism and trustworthiness demonstrated by the content creators. These studies suggest that source cues play an important role in the assessment of perceived credibility. In addition to source cues, social cues also contribute to shaping perceived credibility on social media. Social cues refer to indicators formed by the behaviour or social feedback from other users on social media, such as likes, shares, comments, ratings, recommendations, popularity, and engagement metrics. This heuristic cue type reflects group attitudes and the degree of social endorsement; users are used to employing them as cognitive shortcuts to assess the credibility of information (Sundar, 2008). Within the MAIN model, the technical characteristics of digital media themselves trigger users’ heuristic processing, thereby influencing credibility, attitudes and behaviour. Subsequent research also demonstrated that the heuristic cues that ‘Likes’, ‘Shares’, ‘Retweets’, ‘Comments’ and ‘Followers’ on social media serve as conditions for users to enhance perceived credibility (Lin et al., 2016; Wang et al., 2023). This effect is particularly pronounced in highly homogeneous social media where users engage in shallow cognitive processing (Clayton et al., 2020). However, research findings regarding the effectiveness of social cues remain inconsistent. Some studies suggest that the impact of social validation indicators on credibility assessments is unstable. For example, Kuen and Kuen (2025) found that interactivity has a limited direct impact on the perceived credibility of news, but it may indirectly enhance credibility by strengthening users’ perceptions of source reliability and their trust in digital media. Similarly, Millet et al. (2024) observed that when users prioritise the practicality of the platform and the relevance of the information, for some research, the influence of social cues on perceived credibility is relatively limited.

Within the digital landscape, users find it difficult to systematically verify information on social media and are therefore increasingly reliant on heuristic credibility cues to form rapid judgements of credibility. However, existing empirical findings exhibit significant inconsistencies, making it necessary to systematically integrate the underlying mechanisms. Although previous studies have examined the roles of different types of heuristic cues individually, the literature remains fragmented: most studies focus on a single type of cue, lacking a systematic comparison of the relative efficacy of different heuristic cues; simultaneously, differences in theoretical frameworks, experimental contexts and measurement methods across studies have also made it difficult to reach consistent conclusions. Although meta-analyses have provided an important foundation for understanding online credibility judgement, a mature framework integrating both source-based and social heuristic cues within a social media context has yet to be established. Several meta-analyses examining the role of heuristic cues in perceived credibility have summarised the relationship between the two from various perspectives. These meta-analyses have primarily focused on recent years; Wang et al. (2023) found that certain social cues, such as likes and shares, can significantly influence perceived credibility, although this effect is moderated by contextual factors. Pfänder and Altay (2025), on the other hand, conducted a systematic review and meta-analysis on users’ ability to identify fake news. Their findings indicated that social cues (political congruence) did not have a significant impact on perceived credibility; however, participants exhibited a stronger sceptical attitude towards news items that were politically inconsistent. Furthermore, Zhao et al. (2025) conducted a meta-analysis on the credibility of online health information, systematically identifying multiple factors such as content, source, social interaction, individual characteristics and media empowerment; their findings confirmed the mechanisms underlying credibility assessment in the digital media environment. However, their focus was primarily on the identification of fake news or health information contexts, rather than on social media environments, and did not concentrate on heuristic credibility cues.

In summary, the current literature still lacks a systematic meta-analytic framework that focuses on the social media context and, based on heuristic trust cues, compares the effects of source cues and social cues on perceived credibility, particularly with regard to experimental studies. To address this gap, this study employs a meta-analytic approach to examine the overall effect of heuristic cues on perceived credibility in experimental studies, while identifying potential boundary conditions and sources of heterogeneity. It aims to provide systematic and comprehensive theoretical and empirical evidence to understand how users assess credibility in a social media environment characterised by information overload and misinformation.

This study conducts a meta-analysis of experimental research examining how source cues and social cues influence the perceived credibility of social media content. Specifically, this study aims to address the following research questions:

RQ1: What is the overall effect of heuristic cues on the perceived credibility of social media?

RQ2: Which moderating variables have a significant influence on this effect?

Based on these two research questions, this study aims to examine the influence of heuristic cues on perceived credibility by exploring potential moderating variables, including heuristic cue type, participant type, perceived credibility measurement, experimental setting, sample size, and year of publication, thereby providing a more comprehensive understanding of how users assess credibility in social media environments.

## 2. Methodology

### 2.1. Search Strategy

To identify studies that met the inclusion criteria and to ensure transparency and methodological rigour throughout the research process, the literature search was conducted in accordance with the guidelines for meta-analysis. A comprehensive search was carried out for experimental studies examining the relationship between heuristic credibility cues and perceived credibility on social media, covering six major electronic databases: Web of Science, Scopus, PubMed, PsycINFO, Communication & Mass Media Complete, and Google Scholar, in order to collect as many relevant studies as possible. The search was completed on 10 April 2026. The selected databases covered the fields of communication studies, psychology, public health and interdisciplinary social science research, aligning with the focus of this study.

The search strategy was developed around four core concepts: social media, perceived credibility, heuristic cues, and experimental methodology. Both trust and perceived credibility measures targeting social media content were coded as the outcome of interest and treated as conceptually adjacent indicators for effect size extraction. Boolean operators (AND, OR) and truncation (*) were applied to maximise search sensitivity. To improve transparency and reproducibility, complete search strategies are provided in the Supplementary Materials, including a table with the complete search string, field tags, date range, and applied search limits. Similar search logic was adapted for the remaining databases according to specific requirements. As an example, the full search strategy used for Scopus is presented: ((“social media” OR “social networking site*” OR sns) AND (“perceived credibility” OR credib* OR trust OR believab* OR trustworth*) AND (“heuristic cue*” OR heuristic OR cue* OR “source cue*” OR “social cue*” OR “endorsement cue*”) AND (experiment* OR experimental OR rct)).

### 2.2. Eligibility Criteria

This study adopted the PICOS framework (Population, Intervention, Comparison, Outcome, and Study Design) to define the inclusion criteria, drawing on the Cochrane Handbook for Systematic Reviews of Interventions (Table 1). The inclusion and exclusion criteria were defined according to the following seven aspects. (1) The study population consists of general social media users, including adults and adolescents. Studies focusing solely on users of other media platforms or specific non-user groups will be excluded, unless they provide identifiable, distinct data on general social media users. (2) Only experimental studies will be included. Eligible studies must involve the manipulation of social media heuristic cues and expose participants to different conditions. (3) The comparison condition must be social media content that does not present the manipulated heuristic cues for perceived credibility, serving as the control group. (4) This study includes research examining the impact of manipulated social media heuristic cues on perceived credibility. Studies primarily focusing on other cognitive, emotional, or behavioural outcomes will be excluded, unless they explicitly measure perceived credibility or provide an operational definition of ‘trust’ as users’ evaluative judgement regarding the credibility and reliability of information. (5) Only studies employing one of the following designs will be considered eligible: randomised controlled trials, quasi-experimental designs, or single-group pre-post designs. Survey correlational studies, qualitative studies, and review articles will be excluded. (6) Studies will only be included if they report sufficient statistical information (e.g., means, standard deviations, sample sizes, t-values or *p*-values) to allow for the calculation of effect sizes. Studies lacking sufficient statistical data will be excluded. (7) Only studies written in English meet the inclusion criteria; studies published in other languages will not be considered.

### 2.3. Study Selection

Once the literature search of the database was completed, all retrieved articles were imported into EndNote 20 for management and to remove duplicates. Subsequently, to determine which articles met the inclusion criteria for this study, two independent reviewers (Cao and Norliana) conducted a preliminary screening of the article titles and abstracts, respectively; subsequently, a full-text assessment was carried out on all articles that passed the preliminary screening. Two independent reviewers then examined the full texts against predefined inclusion and exclusion criteria. Studies that did not meet the inclusion criteria were excluded, and specific reasons for exclusion were recorded, including: lack of statistical data required to calculate effect sizes; use of non-experimental research designs; outcome variables irrelevant to this study; or failure to manipulate heuristic credibility cues on social media. Where multiple studies utilised the same sample data, only the study providing the most comprehensive information was retained; where a single study comprised multiple independent experiments, the data set that was most comprehensive and met the inclusion criteria was retained. Both reviewers conducted a detailed review of the full texts; any discrepancies were resolved through discussion and negotiation. Where consensus could not be reached, a third researcher (Saiful) acted as an arbitrator and made the final decision.

### 2.4. Data Extraction and Quality Assessment

Following the completion of the literature screening, this study systematically extracted relevant information from the included studies. Data extracted included author information, publication year, participant type, experiment setting, heuristic cue type, perceived credibility, sample size, and the statistical information required to calculate effect sizes. When extracting the data on perceived credibility, this study did not include them based on specific construct names, but rather judged their inclusion according to their measurement purpose; any outcome measure used to reflect an individual’s evaluation of the perceived credibility (such as perceived credibility, trust, reliability or credibility) was included in the effect size analysis as an outcome variable related to perceived credibility.

We used the QualSyst quality assessment tool developed by Kmet et al. (2004) to assess study quality. It is widely applicable to quantitative research across multiple disciplines and well suited to systematic reviews and meta-analyses in the social sciences and communication studies, providing a comprehensive, transparent and consistent framework for assessing methodological quality. The studies will be scored according to the 14 evaluation criteria established by QualSyst (see Table 2). Each criterion is scored as ‘Yes’ (2 points), ‘Partially met’ (1 point) or ‘No/Not mention’ (0 points); items not applicable are marked as ‘N/A’ and excluded from the scoring. The final assessment was categorised according to the criteria of Kmet et al. (2004) as high quality (≥75%), moderate quality (55–74%) and low quality (<55%). The quality assessment was conducted by two independent reviewers (Cao and Norliana). Disagreements were resolved through discussion, and unresolved cases were referred to a third reviewer (Saiful) for adjudication. Inter-rater reliability was expressed using Cohen’s kappa coefficient. As the included studies primarily employed experimental designs, the QualSyst criteria were used to assess methodological quality. Each assessment item is scored individually; the overall quality score reflects the combined performance across multiple methodological dimensions, rather than any single indicator. During the re-examination process, we confirmed that none of the included studies explicitly reported researcher or participant blinding; consequently, these items were uniformly assigned a score of 0. Although most studies were classified as high quality according to the QualSyst criteria, the absence of reported blinding may have increased the risk of performance and detection bias. Therefore, the overall quality ratings should not be interpreted as indicating the absence of methodological limitations or risks of bias, and the findings should be interpreted with appropriate caution.

### 2.5. Data Analysis

This study used the Comprehensive Meta-Analysis (CMA) software (Version 3) for analysis. With regard to data, studies reported means (M) and standard deviations (SD); effect sizes were calculated directly from these statistics. Where included studies did not directly provide usable means and standard deviations, effect sizes were derived from other statistical information, such as t-values and *p*-values, in order to maximise the inclusion of eligible studies. To ensure statistical independence between effect sizes, only one effect size was extracted from each independent sample. Where a single study reported multiple credibility-related outcome measures using the same sample, only one effect size was retained to maintain statistical independence. Outcome selection followed the predefined criteria described above, and the measure that most closely corresponded to the operational definition of perceived credibility was extracted for analysis. (Borenstein et al., 2021; Lipsey & Wilson, 2001). Specifically, priority is given to those that most closely align with the definition of ‘perceived credibility’ adopted in this review. Similarly, where a single construct was measured using multiple instruments, only one effect size was retained for analysis (Thalheimer & Cook, 2002). No included studies involved shared-control multi-arm comparisons; therefore, no additional adjustment for dependent comparisons was required. Only one effect size was extracted from each independent sample to maintain statistical independence. One included article contained two separate experiments and non-overlapping participant samples, which were treated as independent observations. All effect sizes are calculated with corresponding *p*-values and 95% confidence intervals (95% CI). Given the differences among the included studies in terms of study design, participant characteristics, and experimental settings, this study employs a random-effects model to estimate the overall effect size, taking into account both within-study and between-study variance to account for the possibility that the true effect size may vary across studies. The final results will be assessed according to Cohen’s (1988) criteria, where an effect size of 0.20 is classified as a small effect, 0.50 as a moderate effect, and 0.80 as a large effect. Subsequently, to examine the consistency of effect sizes across studies, heterogeneity was assessed using Cochran’s Q test and the I^2^ statistic. Specifically, Cochran’s Q test is used to determine whether the variation in effect sizes exceeds the range attributable to sampling error, while the I^2^ statistic measures the proportion of total variation attributable to between-study heterogeneity. The results will be interpreted according to the criteria proposed by Higgins et al. (2003), with I^2^ values of approximately 25%, 50% and 75% indicating low, moderate and high heterogeneity.

Subsequently, to further explore the influence of potential moderator variables on the overall effect size, this study conducted subgroup analysis and meta-regression analysis. The moderator variables included in the subgroup analysis comprised heuristic cue type, participant type, perceived credibility measurement, and experimental setting. Meta-regression analyses were performed for sample size and publication year. In addition, a leave-one-out analysis was conducted to assess the sensitivity of the overall results to individual studies to test the robustness of the findings.

We conducted tests for publication bias to examine whether the overall effect size was inflated due to the tendency for ‘significant results to be more readily published’ (Rosenthal, 1979). We assessed publication bias by examining whether the distribution of effect sizes in the funnel plot is symmetrical, followed by a statistical test for potential publication bias using Egger’s regression test (Egger et al., 1997), and trim-and-fill was used to further examine the potential impact of publication bias on the overall effect size. The criteria for judgement will be based on: when the significance level of the intercept term reaches statistical significance (*p* < 0.05), the funnel plot may exhibit asymmetry, suggesting that the study results may be influenced by publication bias; when the test results are not significant (*p* ≥ 0.05), this indicates that no obvious publication bias has been detected. During the interpretation of results, the magnitude and direction of the intercept value are also taken into account to determine the degree of symmetry in the distribution of the effect size by checking whether the intercept is close to 0. At the same time, in order to assess the reliability of the study’s findings, the number of additional studies required to render the overall effect statistically insignificant was also calculated (Lipsey & Wilson, 2001).

## 3. Results

### 3.1. Study Selection Procedure

**Figure 1:**
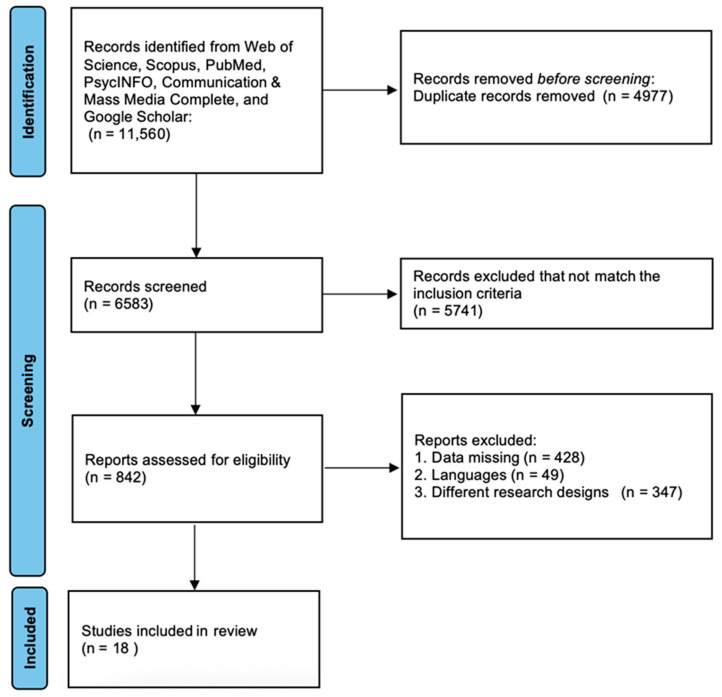
PRISMA Flow Diagram of the Study Selection Process.

During the literature search, we conducted a comprehensive search of six databases relevant to the research topic (Web of Science, Scopus, PubMed, PsycINFO, Communication & Mass Media Complete, and Google Scholar). Using keyword searches based on specific criteria, we identified a total of 11,560 records. After removing 4977 duplicate records using EndNote, the remaining 6583 records proceeded to the title and abstract screening stage. Two independent reviewers (Cao and Norliana) assessed the relevance of these publications against pre-defined inclusion and exclusion criteria. Of these, 5741 publications did not meet the inclusion criteria, leaving a total of 842 papers to proceed to the full-text eligibility assessment stage. Subsequently, during a detailed full-text review, 824 studies were excluded for the following reasons: (1) insufficient information was provided to calculate effect sizes, such as failure to report relevant data or missing data (*n* = 428); (2) articles published in languages other than English (*n* = 49); (3) studies employing research designs that did not meet the requirements of this meta-analysis (e.g., non-experimental designs, qualitative studies, correlational studies; *n* = 347). Ultimately, a total of 18 independent studies met all inclusion criteria and were included in the analysis following a detailed full-text review. The results are shown in Figure 1.

### 3.2. Study Quality Assessment

Two reviewers independently assessed the methodological quality of all included studies using the QualSyst quality assessment tool (Kmet et al., 2004). Inter-rater agreement was measured using Cohen’s κ = 0.77. According to the criteria established by Landis and Koch (1977), this indicates a high degree of agreement between the reviewers, suggesting that the scoring process is highly reliable. The evaluation results showed that 16 studies met high-quality standards, 2 were of moderate quality, and no studies were found to be of low quality. Details of the scores for each study and the scores for each dimension are shown in Table 2. Overall, most studies performed well in terms of the rationality of the study design, sample selection and statistical analysis; however, some studies had certain shortcomings regarding the validation of measurement tools and the control of confounding variables.

### 3.3. Study Characteristics

The meta-analysis included 18 studies involving a total of 14,188 participants. Individual study sample sizes ranged from 80 to 5400 participants. With regard to the types of heuristic cues, the operationalisation of source cues primarily included expert sources, credible sources and official sources; the operationalisation of social cues covered a variety of types, including public opinion environment cues (consensus vs. control), structural cues (interactivity), temporal cues (recency of updates), conformity cues, emotional cues, visibility cues (high visibility vs. low visibility), and engagement ratio cues (like-to-follower ratio). In terms of participant types, 10 studies used student samples, while 8 studies used non-student samples (such as the general public recruited via online platforms). Regarding trust dimensions, 10 studies employed a single-trust-dimension measurement approach, while 8 studies adopted a holistic-dimension measurement approach. In terms of experimental settings, 4 studies utilised offline measurement methods, while 14 studies employed online measurement methods. Detailed characteristics of each study are presented in Table 3.

### 3.4. Effect Size and Homogeneity Testing

**Figure 2:**
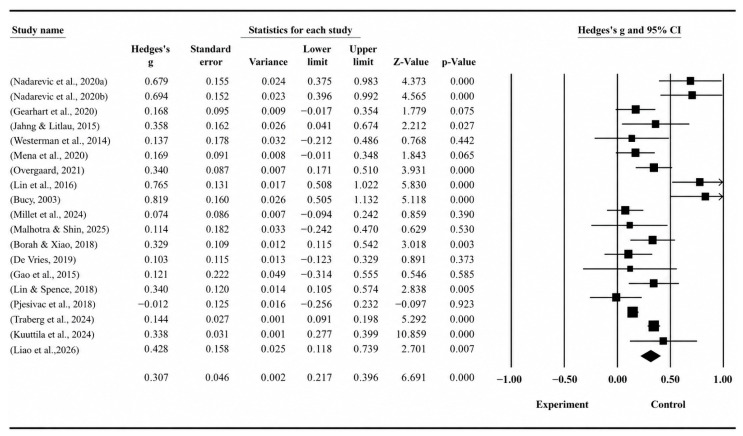
Forest plot for the random-effects model (Nadarevic et al., 2020; Gearhart et al., 2020; Jahng & Littau, 2015; Westerman et al., 2014; Mena et al., 2020; Overgaard, 2021; Lin et al., 2016; Bucy, 2003; Millet et al., 2024; Malhotra & Shin, 2025; Borah & Xiao, 2018; De Vries, 2019; Gao et al., 2015; Lin & Spence, 2018; Pjesivac et al., 2018; Traberg et al., 2024; Kuutila et al., 2024; Liao et al., 2026).

To maintain the statistical independence of the analysis, this study extracted only one effect size from each independent sample. Most of the included studies provided a single effect size. However, as the study by Nadarevic et al. (2020) reported two independent experiments with distinctly separate participant samples and independent experimental manipulations and designs, we treated these two experiments as independent samples in accordance with standard meta-analysis practice. Finally, this study employed a random-effects model to extract data from the 18 eligible studies, ultimately yielding 19 sets of independent effect sizes. The random-effects model was used because it assumes that the true effect sizes across different studies are not entirely consistent; while accounting for inter-study variability, it provides a more generalisable estimate of the overall effect (Borenstein et al., 2021). Table 4 shows the analysis of the 19 independent effect sizes (k = 19) and indicates that heuristic cues have a significant positive effect on perceived credibility, with effect size of Hedges’ g = 0.307 and a 95% confidence interval of [0.217, 0.396], the confidence interval does not cross the zero line, this indicates that the positive effect is statistically significant, and the overall effect test shown as a significant result (Z = 6.691, *p* < 0.001), the perceived credibility in the experimental group was significantly higher than that in the control group. According to Cohen’s (1988) criteria for interpreting effect sizes (0.20 = small effect, 0.50 = medium effect, 0.80 = large effect), the overall effect size in this study represents a positive effect ranging from small to moderate. This suggests that manipulating heuristic cues can enhance perceived credibility of information; however, the extent of this influence is relatively limited and does not constitute a decisive factor. As shown in the figure (Figure 2), there is considerable variation in effect sizes across the studies. In some studies, the confidence intervals straddle the zero value (Gearhart et al., 2020; Mena et al., 2020; Millet et al., 2024), indicating that their effects did not reach statistical significance, whereas other studies demonstrated significant positive effects (Nadarevic et al., 2020; Bucy, 2003; Kuutila et al., 2024). These results suggest that there is high heterogeneity among the included studies, a finding further confirmed by the results of the heterogeneity test, which showed Q(18) = 84.247, *p* < 0.001, and I^2^ = 78.634. Notably, the Q-test reached statistical significance (*p* < 0.001), indicating that the differences in effect sizes observed across studies exceed what can be explained by random sampling error. Furthermore, the I^2^ value reached 78.634. According to the criteria established by Higgins et al. (2003), an I^2^ value exceeding 75 indicates a high degree of heterogeneity. This implies that the impact of heuristic credibility cues on perceived credibility varies across studies and may be influenced by factors such as study design, participant characteristics, cue type, and experiment setting. Consequently, it is necessary to conduct subgroup analysis and meta-regression analysis.

Furthermore, given that Tau^2^ = 0.025 and Tau = 0.158, it is clear that, beyond sampling error, there is indeed a degree of genuine variation in effect sizes across studies. This finding is consistent with the high I^2^ value; therefore, the use of a random-effects model is justified. In conjunction with the results from the forest plot, it is evident that the effect sizes in most studies are positive, indicating that, following the experimental intervention, participants perceived higher levels of credibility than those in the control group. Effect sizes range from g = −0.012 (Pjesivac et al., 2018) to g = 0.819 (Bucy, 2003), indicating significant variation across studies. Studies with larger effect sizes include: Bucy (2003), g = 0.819; Lin et al. (2016), g = 0.765; and two independent effect sizes were extracted from Nadarevic et al. (2020) (g = 0.679 and g = 0.694). In contrast, some studies reported weaker or even non-significant effects, such as: Pjesivac et al. (2018), g = −0.012; Millet et al. (2024), g = 0.074; and Malhotra and Shin (2025), g = 0.114; although individual studies failed to find significant effects, overall, most effect sizes lie to the right of the zero line.

Overall, these results suggest that exposure to social media content (such as information manipulated through source cues or social cues) can significantly enhance users’ perceived credibility of that content, albeit to a limited extent.

### 3.5. Sensitivity Analysis

In this analysis, a sensitivity analysis was conducted by recalculating the pooled effect size after sequentially excluding each individual study. The results indicate that, following the sequential exclusion of each study, the pooled effect size remained statistically significant (all *p* < 0.001), and the estimated pooled effect size ranged from 0.284 to 0.325, showing high consistency with the pooled effect size from the primary analysis (g = 0.307, 95% CI [0.217, 0.397]). Specifically, after excluding any single study, the range of the pooled effect estimates was 0.284 to 0.325, with a maximum deviation of approximately ±0.023 compared to the main analysis effect size of 0.307; furthermore, the lower limits of all recalculated confidence intervals were above 0.217. These findings indicate that the meta-analysis results demonstrate good robustness, with no single study exerting an excessive influence on the overall effect size.

### 3.6. Publication Bias

**Figure 3:**
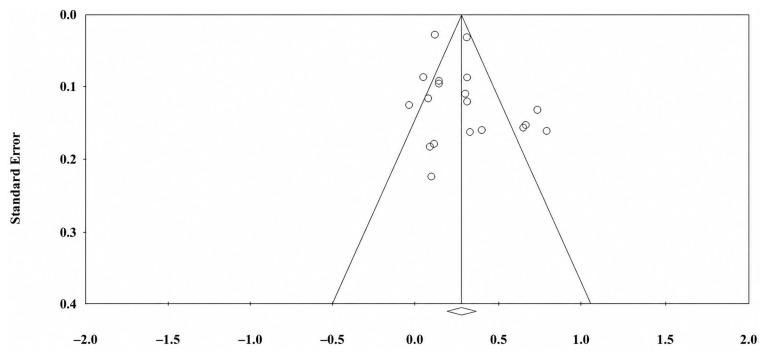
Funnel plot of the overall mean effect size analysis.

To determine whether the included studies systematically overestimated or underestimated the true effect size, this study further tested for publication bias. The intercept test in the Egger test yielded an intercept value of 1.05266, with *p* = 0.197 > 0.05, indicating that no strong evidence of publication bias was detected; the difference between the intercept value and zero was not statistically significant. Furthermore, the t-value was 1.34395. This small t-value further supports the conclusion that there is no strong evidence of publication bias. Furthermore, this study employed the trim-and-fill method to conduct a robustness test for publication bias. Under a random-effects model, and assuming that the potentially missing studies were located to the left of the mean, the estimated number of missing studies was found to be 0. The overall effect size remained consistent before and after adjustment (before adjustment: g = 0.307, 95% CI [0.217, 0.397]; after adjustment: g = 0.307, 95% CI [0.217, 0.397]), indicating that no bias in the effect size due to potential missing studies was detected, and that the study conclusions remain robust following correction for publication bias. Additionally, the results of the classic fail-safe N test indicated that the number of missing studies (the Fail-Safe factor) was 856.00000. This implies that 856 unpublished studies showing ‘no significant effect’ would need to be added. This figure exceeds 5k + 10 (5 × 19 + 10 = 105) and is far greater than the critical value of 105, indicating that the results of the available evidence did not indicate strong evidence of publication bias. The funnel plot (Figure 3) visually displays a roughly symmetrical distribution, corroborating the results of the quantitative analysis. The majority of studies (19 data points) are distributed between 0.0 and 1.0 with no obvious asymmetry. Although the effect sizes of individual small-sample studies fluctuate slightly, the overall pattern did not reveal any significant publication bias. Therefore, the conclusions of this study are reasonably robust.

### 3.7. Subgroup Analyses

To investigate potential sources of heterogeneity among the included studies and to examine whether the magnitude of the overall effect varies according to study characteristics and experimental conditions (Borenstein et al., 2021), we conducted subgroup analyses based on several theoretical and methodological moderators. Specifically, the included studies were categorised according to the type of heuristic cue (source-based cues vs. social cues), participant type (student samples vs. non-student samples), experimental context (online vs. offline), and the measurement dimensions of trust (unidimensional vs. multidimensional) to examine whether the effect of heuristic credibility cues on perceived credibility varies across different conditions and sample characteristics. The analysis employed the Q-statistic under a random-effects model to assess between-group differences (Hedges & Vevea, 1998), and subgroup analyses were conducted only in categories with a sufficient number of studies to ensure the validity of the results (Borenstein et al., 2021). The relevant results are presented in Table 5.

#### 3.7.1. Heuristic Cue Type

Different types of heuristic cues may influence individuals’ judgement of credibility through distinct psychological mechanisms. This study conducted a subgroup analysis of the differences in effects between source cues and social cues to examine whether there are differences in the impact of different types of heuristic cues on perceived credibility. The results of the subgroup analysis indicate that the type of heuristic cue is a significant moderator of perceived credibility (QB = 7.815, *p* = 0.005). Specifically, the combined effect size for the 11 source cues was g = 0.406 (95% CI [0.276, 0.536]), representing a moderate effect size; the combined effect size for the 8 social cues was g = 0.185 (95% CI [0.100, 0.269]), indicating a small effect size.

#### 3.7.2. Participant Type

As participants may differ in terms of media usage experience, digital literacy and information processing styles, participant type may influence the effect of heuristic cues on perceived credibility. The study examined and compared the differences in effects between student and non-student samples. The results indicate that this variable exerts a substantial moderating effect (Q_B_ = 4.397, *p* = 0.037). Furthermore, the effect size for the 10 studies involving student groups reached a moderate level (g = 0.427, 95% CI [0.245, 0.610]), while the 9 studies involving non-student groups showed only a small effect (g = 0.210, 95% CI [0.119, 0.300]). The results suggest that the effect size for the student group was significantly larger than that for the non-student group. Both the student group (I^2^ = 75.558) and the non-student group (I^2^ = 73.906) exhibited high levels of heterogeneity.

#### 3.7.3. Perceived Credibility Measurement

As a complex construct, perceived credibility has been measured across different dimensions in existing research, which may result in varying degrees of capture of perceived credibility and consequently affect estimates of the heuristic cue effect. Therefore, the studies were categorised into two groups: those using multidimensional measurements and those using unidimensional measurements. Subgroup analysis results indicate that differences in effect sizes between different levels did not reach statistical significance (Q_B_ = 0.424, *p* = 0.515), suggesting that this variable does not exert a significant moderating effect. In the mixed-effects analysis, multidimensional measures (8 studies) yielded g = 0.350, [0.169, 0.530], representing a small to moderate effect. For unidimensional measures (11 studies), g = 0.280, 95% CI [0.172, 0.388], representing a small to moderate effect; heterogeneity was high in both groups (multidimensional I^2^ = 84.665, unidimensional I^2^ = 67.162).

#### 3.7.4. Experiment Setting

Differences in experimental settings may affect the effectiveness of heuristic credibility cues; this subgroup investigated whether there are differences in the effectiveness of heuristic credibility cues between online and offline experimental settings. The exploratory analysis suggested that the moderating effect of experimental setting design was not statistically significant (Q_B_ = 2.980, *p* = 0.084), falling short of the conventional level of statistical significance (*p* < 0.05). At the same time, the pooled effect size for offline experiments (4 studies) (g = 0.530, 95% CI [0.228, 0.833]) remained larger than that for online experiments (15 studies) (g = 0.254, 95% CI [0.168, 0.340]). Heterogeneity was high in the online group (I^2^ = 74.135) and the offline group (I^2^ = 75.686); this suggests that there may be other moderating variables that influence the relationship between heuristic cues and perceived credibility.

### 3.8. Meta Regression

To further investigate the potential sources of heterogeneity among the 19 effect sizes, this study employed meta-regression analysis. A weighted linear method based on a hypothetical statistical model, which extends the random-effects model by analysing the influence of latent moderator variables (Thompson & Higgins, 2002). This method is particularly suitable for situations where the moderator variable is continuous, such as year of publication, sample size or participants’ mean age (Sánchez-Meca & Marín-Martínez, 1998). Meta-regression analyses were conducted to examine whether publication year and sample size moderated the observed effect sizes.

#### 3.8.1. Sample Size

To examine whether sample size could significantly predict the variability in effect sizes across studies, we conducted a random-effects meta-regression analysis. The results (Table 6) indicated that sample size did not significantly moderate the relationship between heuristic credibility cues and perceived credibility (β = −0.0000, SE = 0.0000, Z = −0.86, *p* = 0.387, 95% CI [−0.0001, 0.0000]). The overall regression model was also non-significant (Q = 0.75, df = 1, *p* = 0.387), indicating that variation in sample size does not account for the heterogeneity across studies. Furthermore, after including sample size as a covariate in the model, significant residual heterogeneity persisted (I^2^ = 77.72%, Q = 76.29, *p* < 0.001), suggesting that other uninvestigated moderating variables may account for the observed variability in effect sizes. Compared with the null model, the proportion of between-study variance explained by the regression model was very small (R^2^ analogueue = 0.00), further illustrating that sample size contributes little to the model’s explanatory power. This indicates that heuristic cues have a relatively stable effect on perceived credibility across studies with different sample sizes.

#### 3.8.2. Publication Year

To investigate potential sources of heterogeneity across studies, this study conducted a random-effects meta-regression analysis using year of publication as the predictor variable. The results show (Table 6) that publication year is significantly negatively correlated with effect size (β = −0.0208, SE = 0.0093, *p* = 0.025), and the regression model is significant overall (Q = 5.00, *p* = 0.025), indicating that publication year accounts for a considerable portion of the heterogeneity across studies. The negative coefficient implies that, compared with earlier studies, the credibility effect reported in recent studies shows a decreasing trend, suggesting that the influence of heuristic cues on perceived credibility is weakening. Despite the inclusion of publication year, the model still exhibits significant residual heterogeneity (I^2^ = 75.85%, *p* < 0.001), and publication year accounts for only approximately 12% of the variance between studies (R^2^ analogue = 0.12), indicating that additional moderators may contribute to the remaining heterogeneity. Overall, the influence of source and social cues on perceived credibility has diminished over time, reflecting the evolution of the social media environment and users’ increased awareness of credibility cues.

## 4. Discussion

This meta-analysis comprehensively examined the effect of social media source cues and social cues on users’ perceived credibility. The results indicate that heuristic credibility cues were associated with a statistically significant increase in users’ perceived credibility of information (g = 0.307, 95% CI [0.217, 0.396], *p* < 0.001). Furthermore, this effect varied across different moderating variables, including the type of heuristic cue, participant type, trust dimension, experimental setting, sample size and year of publication.

### 4.1. Effect of Heuristic Credibility Cues on Perceived Credibility

In the information-saturated environment of social media, users tend to rely on heuristic cues to judge the credibility of information; among these, source cues exert a greater influence than social endorsement cues, and this effect is particularly pronounced among students. This meta-analysis synthesised 18 experimental studies (N = 14,188), finding that exposure to social media content significantly enhanced users’ perceived credibility (g = 0.307, *p* < 0.001). Although the effect sizes were small to moderate, this finding remains of statistically significant theoretical and practical importance in a digital environment characterised by fragmentation and cognitive overload. Given that users have relatively limited attention spans and time when accessing information through social media, making it difficult for them to examine content in depth, they tend to rely on heuristic cues rather than systematic information processing strategies (Chaiken et al., 1989). They use heuristic cues such as source labels, engagement metrics, timeliness signals and interactivity as cognitive shortcuts to judge the credibility of information. Empirical research has demonstrated that, among these heuristic cues, those indicating expert or official sources can significantly enhance users’ perceived trust (Nadarevic et al., 2020; Borah & Xiao, 2018; Lin & Spence, 2018; Traberg et al., 2024; Lin et al., 2016; Bucy, 2003). Social cues (such as likes, shares and comments), as major heuristic cues, also affect users’ perceived credibility (Jahng & Littau, 2015; Westerman et al., 2014; Mena et al., 2020; Overgaard, 2021; Pjesivac et al., 2018; De Vries, 2019). At the same time, with the rapid development of algorithmic recommendations, influencer livestreams and AI-generated content (AIGC), heuristic cues may further blur the boundaries between expertise, popularity and authenticity (Millet et al., 2024; Malhotra & Shin, 2025). The results of this meta-analysis also indicate that heuristic cues generally contribute to users’ perceptions of information credibility, and that perceived credibility is indeed influenced by the combined effect of social media source cues and social cues. As these types of cues may involve different psychological mechanisms, the overall effect observed in this study should be interpreted as the average effect of different heuristic credibility cues across heterogeneous contexts, rather than as evidence that all cues operate according to a uniform or consistent mechanism. Given the variation in cue operationalisation, participant characteristics, and experimental settings across studies, caution is warranted when generalising the observed effect to all social media environments.

#### 4.1.1. Heuristic Cue Type

An analysis of the impact of different heuristic cue types on perceived credibility revealed that source cues and social cues yielded different pooled effect estimates (Q_B_ = 7.815, *p* = 0.005). The effect size for source cues (g = 0.407) was significantly higher than that for social cues (g = 0.185), indicating that users rely more heavily on cues related to the source of information when assessing its credibility. This finding is consistent with Source Credibility Theory, which emphasises the crucial role of expertise and authority in shaping perceptions of information credibility. Social cues also exerted a positive influence, though their impact was relatively weaker compared to source cues. This may reflect users’ growing wariness regarding the potential manipulation of common social cues, such as the number of likes and shares, particularly given the increasing prevalence of bot accounts, paid reviews and algorithmic recommendations. This finding supports the result in the study by Ferrara et al. (2016), which suggests that public perceptions of information popularity and credibility are being influenced by interaction data generated by bots.

At the same time, the findings of this study differ somewhat from those that emphasise the persuasive power of social cues. Although some previous studies have shown that users typically regard a high number of likes and shares as signals of collective endorsement and the validity of information (Sundar, 2008; Wang et al., 2023), particularly during the early stages of social media development, relevant research has suggested that the influence of peer endorsement may surpass that of traditional authoritative sources, and indicated that this phenomenon is particularly pronounced among younger user groups (Djafarova & Rushworth, 2017; Sundar, 2008). However, the present study found that the effects of social cues were, on the whole, relatively weak; a plausible explanation may be that their persuasive power is waning in the contemporary digital environment. This may be due to the public’s growing awareness that engagement metrics on social media are subject to manipulation. Issues such as bot accounts, fake followers and algorithmic amplification have eroded users’ trust in metrics such as likes, shares and follower counts. The exposure, during major political and public health events, of practices that artificially amplify signals of social endorsement to create the illusion of public consensus (Allcott & Gentzkow, 2017; Woolley & Howard, 2018) has further eroded users’ perceived trust. Existing research indicates that manipulated engagement metrics do indeed influence users’ judgements of perceived credibility (Ferrara et al., 2016; Cinelli et al., 2020).

On the other hand, the emergence of this situation may be linked to the development of social media systems. Specifically, as algorithmic recommendation systems gradually replace traditional information-seeking habits, users engage in fewer active information searches and are increasingly exposed to information sources outside their personal social circles; consequently, they rely more heavily on source cues such as verification badges, institutional affiliations and expert status when assessing the credibility of information (Metzger et al., 2010). This trend is particularly pronounced in high-stakes information contexts, especially in the fields of health communication, political communication and crisis reporting (Flanagin & Metzger, 2007; Lucassen & Schraagen, 2013). For example, during the COVID-19 pandemic, accounts belonging to official bodies such as the World Health Organisation were generally perceived as more credible than user-generated content with high engagement metrics (Cinelli et al., 2020). Furthermore, the diminishing effect of social cues may also reflect improvements in users’ digital media literacy and social media experience. Users may maintain a higher degree of scepticism towards mere popularity metrics and adopt selective heuristic processing strategies. This explanation is consistent with findings from related research, which indicate that heuristic processing is influenced by factors such as prior knowledge, digital literacy and platform usage experience (Metzger & Flanagin, 2013).

To summarise, although different types of heuristic cues continue to influence users’ perceptions of credibility, their impact may vary depending on specific experimental conditions and social media contexts; consequently, the observed subgroup differences should be interpreted with caution and regarded as evidence of potential differences rather than conclusive proof of a stable effect of specific cues.

#### 4.1.2. Participant Type

Following a subgroup analysis of participant types, the results showed a statistically significant overall effect (Q_B_ = 4.367, *p* = 0.037), with the effect size for the student sample (g = 0.427) being statistically higher than that for the non-student sample (g = 0.210). This may be because the student group uses social media more frequently; greater usage implies that, in terms of perceived trust, students are more inclined to rely on heuristic processing strategies to improve efficiency. Although they are relatively familiar with digital technology, their capacity for critical information verification is limited, making them more susceptible to the influence of heuristic cues. In contrast, the non-student group possesses richer real-life experience, more time, and more diverse information-gathering habits. Nevertheless, heuristic cues still exerted a significant influence on the non-student group, suggesting that the difference between the two groups lies primarily in the degree of influence rather than the direction of influence. It can therefore be observed that, due to the frequent use of social media and continuous exposure to vast amounts of online information, young users and students tend to rely more on heuristic processing strategies when evaluating online content (Metzger et al., 2010). At the same time, although students generally possess a high level of digital literacy and technical proficiency, technical ability is not synonymous with information evaluation ability. Particularly in situations of information overload, students often rely on cues such as the identity of the information source, verification badges, the number of likes, and the number of comments to help them make rapid judgements about credibility. This behaviour is consistent with the theoretical perspectives of the Heuristic-Systematic Model (HSM) and the Limited-Capacity Model of Information Processing. Wineburg and McGrew (2019) point out that while young users are familiar with the operation of digital platforms, they do not necessarily possess the corresponding critical information verification skills; indeed, high levels of digital engagement may actually reinforce their reliance on rapid information processing. Consequently, students’ sensitivity to heuristic cues reflects their long-term adaptation to an information consumption model characterised by ‘skimming’ and ‘instant judgement’.

At the same time, other studies have put forward the opposite view, suggesting that young users, owing to their higher digital literacy and familiarity with manipulative mechanisms such as algorithmic recommendations and fake interactions, may exhibit a stronger sceptical attitude towards online information (Guess et al., 2019; Vraga & Tully, 2021). The discrepancy between the present findings and previous studies may be explained by the situational dependence of heuristic processing. In an experimental setting, participants are presented with simplified information stimuli and limited contextual information, making them more likely to rely on the most intuitive heuristic cues. As students have long adapted to platform environments such as short videos and news feeds, they are more accustomed to rapid scanning and filtering based on limited cues; This may explain why students are more likely to rely on heuristic cues in controlled experimental contexts. Moreover, student samples tend to be relatively homogeneous with respect to age, educational background, and media usage patterns, which may increase the observed effect size of heuristic cues. By contrast, non-student samples are typically more diverse and engage in more complex information judgement processes. Although the heuristic effect remains significant, the effect size is markedly smaller than that observed in the student group. However, given the high heterogeneity between studies and the relatively limited evidence available for participant type, these findings should be interpreted with caution and regarded as exploratory findings.

#### 4.1.3. Perceived Credibility Measurement

As research on social media credibility exhibits significant variations in the measurement of perceived credibility, with some studies employing unidimensional measures whilst others use scales covering multiple dimensions, we conducted a subgroup analysis of perceived credibility measurements. The results indicated that both groups exhibited positive effect sizes. However, studies employing multidimensional scales to measure perceived credibility yielded a larger effect size (g = 0.350), whereas those using unidimensional measures produced a relatively smaller effect size (g = 0.280). Consequently, the difference between these two measurement approaches did not reach statistical significance (Q_B_ = 0.424, *p* = 0.515). These findings suggest that the influence of heuristic cues on perceived credibility appears to follow a relatively consistent pattern across different measurement methods (Appelman & Sundar, 2016; McCroskey & Teven, 1999; Metzger et al., 2010). Consequently, the results of this study are more likely to reflect the fact that heuristic cues can influence users’ overall evaluation of the credibility of information. However, given the limited existing evidence and differences in the operational definitions of perceived credibility across studies, caution is still required when interpreting these results as indicating equivalence between different dimensions of credibility.

Whether one-dimensional or multi-dimensional measurement methods are used, heuristic cues trigger users’ overall perception of credibility, and their effects are generally consistent. This may be because perceived credibility can be measured as a multidimensional construct, but each dimension contributes to the user’s overall assessment (Westerwick, 2013; Metzger & Flanagin, 2015). Consequently, the impact of heuristic cues on perceived credibility does not differ significantly depending on whether a study employs one-dimensional or multi-dimensional measurements; this implies that future research may select the appropriate measurement method for assessing perceived credibility based on specific research objectives. Specifically, if the research objective is to conduct a rapid assessment or a large-scale survey, a unidimensional measurement is sufficient to effectively reflect the level of perceived credibility; if the objective is to gain an in-depth understanding of the multi-layered structure of credibility, a multidimensional measurement will yield richer and more comprehensive research findings.

#### 4.1.4. Experiment Setting

To assess the potential moderating role of the experimental setting, this study conducted a subgroup analysis, the results of which indicated that the moderating effect of this variable was not significant (*p* = 0.084). A comparison of the two experimental settings suggested that the pooled effect size was numerically larger in offline experiments (e.g., Bucy, 2003; Westerman et al., 2014); larger effect sizes are typically observed, with an effect size (g = 0.530) higher than that in the online experimental setting (g = 0.254). One possible explanation is that offline experiments provide a more controlled environment, making it easier for participants to notice the heuristic cues manipulated by the researchers. Enhanced control over the manipulation process may amplify the effectiveness of source and social cues, thereby generating a larger effect size. This tentative pattern is broadly consistent with the heuristic-systemic model (Chaiken, 1980), which posits that individuals’ reliance on heuristic cues may vary across different contextual conditions and cognitive processing demands. However, the existing evidence remains insufficient to support the view that experimental context exerts a consistent moderating effect. In particular, a large number of experimental studies on social media have generally employed platform interfaces and experimental materials with high simulation quality for manipulation; the gap in ecological validity between experimental contexts is narrowing, thereby weakening the role of experimental context as a moderating variable (Javed et al., 2024). Furthermore, the number of studies included in each category was limited; there were only four studies (k = 4) conducted under online experimental conditions, which weakened the statistical power of subgroup comparisons and increased the uncertainty in the estimates of the pooled effects. At the same time, there was a high degree of heterogeneity within each subgroup, suggesting the possible presence of unidentified moderating factors. The results indicate that the moderating effect of the experimental setting did not reach statistical significance; however, the observed trend suggests that the experimental setting may act as a moderator of credibility perception. This potential boundary condition warrants further exploration in future research. Consequently, the interpretation of the moderating effects and related analytical findings in this study should be approached with caution; they should be regarded as exploratory findings rather than definitive conclusions. Future research could further investigate and clarify this moderating mechanism by increasing the number of studies and incorporating a wider range of experimental settings.

#### 4.1.5. Sample Size

To examine whether study size influences estimates of effect sizes, this study included sample size in a meta-regression analysis. The results indicate that sample size has no significant effect on the impact of heuristic cues on the perceived credibility of information (*p* = 0.3916); this study did not identify a systematic relationship between sample size and the observed effect size. However, given the relatively limited number of effect sizes included in the study and the high level of heterogeneity between studies (I^2^ = 77.71%), this result should not be interpreted as conclusive evidence that sample size has no effect. From a theoretical perspective, this finding is consistent with the view held in relevant literature that heuristic processing is a commonly occurring mode of information processing, the functioning of which may not depend entirely on study size (Chaiken, 1980). Overall, the findings of this study suggest that sample size does not explain the heterogeneity observed across studies; other characteristics, such as the type of heuristic cue, participant type and experimental setting, may play a more significant role in the formation of effect differences and warrant further investigation in future research. Consequently, given the limited number of included studies and the high level of between-study heterogeneity, the findings of this study regarding the moderating role of sample size are best regarded as exploratory evidence and a basis for generating future hypotheses, rather than as definitive conclusions regarding the impact of sample size on effect sizes.

#### 4.1.6. Publication Year

A statistically significant negative relationship was found between publication year and the effect of heuristic cues (β = −0.0208, *p* = 0.0254), suggesting that the effect size declines over time since publication. One possible explanation is that, as the social media environment continues to evolve, users’ exposure to misinformation, commercial promotions and algorithmically recommended content has increased, which may prompt them to adopt more cautious strategies when assessing credibility. This may suggest a reduced reliance on traditional heuristic cues (Traberg et al., 2024; Kuutila et al., 2024; Millet et al., 2024); secondly, improvements in methodological rigour and an emphasis on reproducibility may also lead to more conservative estimates in recent studies. Nevertheless, even after controlling for the year of publication, heterogeneity remains high (I^2^ = 75.85), suggesting the presence of other unmeasured moderating variables. The results of the meta-regression analysis indicate that, in more recent studies, the effects of source cues and social endorsement cues on perceived credibility are relatively weaker. Several studies on users’ perceptions of fake news, algorithmic manipulation and inauthentic collaborative behaviour suggest that users are becoming increasingly suspicious of information on social media (Lazer et al., 2018; Humprecht et al., 2020; Ferrara et al., 2016; Woolley & Howard, 2018). This phenomenon may be linked to the opening up of information sources and the weakening of traditional gatekeeping mechanisms (Lazer et al., 2018); at the same time, as users’ information literacy improves and concerns about the potential manipulation of social validation cues intensify, they have adopted a more cautious attitude towards social media (Humprecht et al., 2020). Although early social media research indicated that social cues such as ‘likes’, shares and verification badges were typically regarded as signals of credibility (Westerman et al., 2014), repeated exposure to misinformation has eroded perceived credibility in these superficial cues. As platforms increasingly rely on algorithmic recommendation systems, users are exposed to a greater volume of unfamiliar information sources, anonymous accounts and commercial promotional content, further exacerbating this trend. Coupled with users’ growing awareness of platform governance and surveillance capitalism (Zuboff, 2019), their trust in information sources and social cues has declined further. Consequently, users may gradually reduce their reliance on single heuristic cues and incorporate more dimensions of information when assessing credibility (van Dijck et al., 2018; Couldry & Hepp, 2017). In addition, the influence of heuristic cues is not limited to static evaluations; it is also embedded within the mechanisms of social media dissemination. For example, monitoring studies based on real social media data have found that misinformation often gains greater visibility through emotional expressions, high levels of engagement or a large number of shares (Sufi et al., 2022; Aygün et al., 2021). This suggests that cues of social approval, such as ‘likes’ and shares, may further reinforce users’ perceptions of an information item’s credibility through algorithmic amplification and group dissemination. Furthermore, ongoing refinements and advances in research methodology (such as pre-registration, larger sample sizes and more realistic experimental settings) may have led to more conservative estimates of effect sizes, which partly explains the downward trend observed in these estimates.

Overall, the results of the publication year analysis explained a small portion of the heterogeneity, and the impact of heuristic cues may also depend on specific contextual factors, such as different social media platforms, subject area, artificial intelligence, and political or cultural contexts. It should be noted that the results of the meta-regression regarding publication year should be regarded as exploratory findings rather than definitive evidence; the publication year may simultaneously reflect changes in multiple aspects, including the platform environment, research design, measurement tools and sample characteristics. Future research could further incorporate more specific variables to investigate these potential mechanisms.

## 5. Limitations and Future Research

This study is subject to several limitations, despite providing a comprehensive meta-analytic synthesis of experimental research on the effects of heuristic cues on perceived credibility. For the number of studies included, only 18 experimental articles were included in the present review, as some subgroups were represented by a very small number. Consequently, the statistical power and stability of the moderator analyses may be limited, due to the small effect sizes and significant heterogeneity in the subgroup analysis, the findings regarding moderation effects are exploratory only and require validation in studies with larger sample sizes, the high overall heterogeneity suggests that there are still underlying factors that have not been fully accounted for, and for effect size extraction, these constructs differ conceptually, combining them may have introduced some conceptual heterogeneity, which constitutes a limitation of this study. Future research should therefore examine additional moderators that may influence the relationship between heuristic cues and perceived credibility, because of several potentially relevant moderators could not be examined in the present meta-analysis, there is scope for further refinement in the categorization of subgroup variables; meanwhile, the present research was unable to distinguish in depth between differences across social media platforms and cross-cultural contexts, different platforms exhibit significant differences in terms of information presentation, interaction mechanisms, algorithmic recommendation logic, and the design of social cues; moreover, the platform ecosystems and media usage habits in different countries and regions may also influence users’ credibility judgement processes, with the increasing proliferation of AI-generated content, deep-fakes, and algorithm-manipulated dissemination, the mechanisms underlying traditional source cues and social cues may also undergo changes. Future research could conduct more in-depth comparisons of the mechanisms underlying social media credibility formation by integrating different platforms, cross-cultural contexts, and AI-driven communication environments. Finally, the analysis reports were conducted using Comprehensive Meta-Analysis (CMA) software; although it provides relatively stable effect size estimates, it was not possible to address the issue of effect size dependence using a multilevel model. Future research could employ multilevel meta-analysis methods to obtain more accurate results regarding the effects of heuristic cues on perceived credibility. In addition, although a variety of complementary diagnostic methods have been employed for the assessment of publication bias, including Egger’s test, trim-and-fill, fail-safe N and funnel plots, high heterogeneity may reduce the sensitivity of these diagnostic methods; in particular, when the number of included studies is relatively small, Egger’s test may lack sufficient statistical power.

Despite the limitations outlined above, this study provides a systematic meta-analysis of experimental research into the effects of heuristic cues on perceived credibility. The findings contribute to a deeper understanding of the mechanisms underlying users’ judgement of credibility and establish a crucial foundation for future research into the mechanisms of credibility assessment within more complex and diverse social media environments.

## 6. Conclusions

This study evaluated the association between heuristic cues and perceived credibility through a systematic meta-analysis, focusing in particular on the role of the two main types of heuristic cues, source cues and social cues, in shaping perceived credibility. The results indicate that within included studies, heuristic credibility cues are generally associated with higher perceived credibility (g = 0.307, *p* < 0.001), with effect sizes ranging from small to moderate; however, this relationship varies significantly across different research contexts.

Subgroup analyses were also conducted to explore the potential influence of other variables. Among these, the type of heuristic cue and participant type significantly moderated the overall effect. Specifically, Source cues exhibited a relatively larger average effect on perceived credibility than social cues in the included studies, suggesting that in the context of the present study, source cues associated with expertise or authority are more likely to be taken into account when assessing perceived credibility. In contrast, although social cues, primarily metrics such as likes, comments and shares, also enhanced users’ perceived credibility, their influence was relatively weaker. This result reflects that there has been growing concern within the user community regarding the manipulability of social interaction metrics, but this mechanism still requires further verification. Future research could further explore whether the cognitive mechanisms underlying social media users’ perceptions of credibility change in different platforms and content types of the AI environment, addressing two key questions: whether source cues continue to exert an effective influence on credibility assessment, and whether the effectiveness of social endorsement cues varies significantly depending on individual digital literacy and critical evaluation skills. Furthermore, a relatively higher average effect was observed in the student groups compared to non-student groups, indicating that students were more susceptible to the influence of heuristic cues; however, the specific mechanisms underlying this difference could not be clearly identified in this study. Future research could systematically examine factors such as age, intensity of social media use, information literacy, and familiarity with the digital communication environment to determine which factors account for these group differences. The meta-analysis results further indicate that the effect sizes are relatively small compared with those in more recent research for the effect of heuristic cues on perceived credibility. This trend may be linked to changes in research contexts, platform environments and methodology; for example, the public’s growing vigilance regarding misinformation, algorithmic manipulation and AI-generated content may lead users to become more cautious on social media. These findings suggest that the process of assessing perceived credibility may depend on particular circumstances.

In contrast, perceived credibility measurement, experimental setting and sample size did not exhibit significant moderating effects. However, these factors may still influence perceived credibility under specific conditions, particularly in the context of AI technology use, differences in social media features, users’ extensive media usage experience, and improved media literacy. Future research could further examine the impact of AI-generated content (AIGC), algorithmic recommendation systems, and social media dissemination mechanisms on users’ assessment of perceived credibility through heuristic cues, while exploring the distinctive characteristics of heuristic credibility cue processing across different platform and cultural environments.

In practical terms, this study offers important insights for social media platforms to refine their dissemination mechanisms and optimise their functionalities. This implies that social media platforms such as Facebook, Instagram, TikTok and X (formerly Twitter) should carefully consider the potential impact of different heuristic cues when designing a credibility indication mechanism. Furthermore, this is particularly important when sensitive information relating to politics, science or health appears on social media. As the analysis clearly demonstrates, source cues showed a relatively larger average effect than social cues in the included studies, indicating that in certain research settings official status continues to wield significant influence in the digital environment. News organisations, governments and public health bodies can similarly enhance perceived credibility through strategies such as transparency, the display of sources, and official and expert endorsements. It should be noted, however, that misinformation can also expand its reach through the forgery of certification badges, the exaggeration of engagement metrics, fake comments or the fabrication of expert identities. Consequently, social media platforms should be cautious of misleading credibility cues, particularly given that students are more susceptible to the influence of heuristic cues than non-students; there is a need to improve users’ media literacy. At the same time, social media platforms should guide users to critically evaluate heuristic cues and help them identify manipulated signals. This is especially crucial against the backdrop of the increasing prevalence of generative artificial intelligence, where users find it difficult to assess AI-generated content. Consequently, future social media platforms must carefully manage the relationship between heuristic credibility cues, AI-generated content and algorithmic recommendations.

The findings expand the theoretical understanding of heuristic processing in digital communication environments, while highlighting the need for future research to delve deeper into the interplay between AI environments and variations in user literacy, thereby enabling a better understanding of the mechanisms underlying the presentation of heuristic cues on social media. Overall, this study supports the existence of a positive association between heuristic credibility cues and perceived credibility; however, this relationship is not stable or consistent, but is jointly influenced by heuristic cue type, participant type, experiment setting, and other unmeasured factors, given the high level of heterogeneity, the limited number of studies in some subgroups, and the limitations of the meta-analysis, these findings should be interpreted as exploratory evidence rather than universal principles applicable to all social media contexts. Future research needs to further identify the specific boundary conditions under which heuristic credibility judgements come into play. Future research could employ multilevel meta-analytic models to account for the nested structure of multiple effect sizes within a single study, thereby reducing estimation bias that may arise from the non-independence of effect sizes. A more refined classification of heuristic cues could be developed to identify more accurately which specific cues drive credibility judgements. Future research should also conduct platform-specific analyses to examine differences arising from variations in information presentation, algorithmic recommendation mechanisms and interaction structures. Furthermore, robust variance estimation or multivariate meta-analysis could be employed to address the issue of effect size dependence.

## Figures and Tables

**Table 1:** Inclusion and Exclusion Criteria According to the PICOS Framework.

Parameter	Inclusion Criteria	Exclusion Criteria
Population	Actual social media users or ordinary internet users can provide explicit assessments of perceived credibility.	Other media users, as well as specific non-user groups.
Intervention	Exposure to manipulated heuristic credibility cues (e.g., source cues, social cues) on social media	No manipulation of heuristic credibility cues
Comparison	A valid control condition involving exposure to unmanipulated social media content (without heuristic credibility cues)	No appropriate control group, or only comparisons between different types of manipulated cues
Outcome	Perceived credibility/trust of social media information	Other assessments (e.g., attitudes, sharing intention, behaviour)
Study design	Randomised controlled trials, quasi-experimental designs, or single-group pretest–posttest designs	Non-experimental studies, Qualitative research, correlational studies, surveys or review studies
Study data	Studies must report sufficient data to calculate effect sizes (e.g., means, standard deviations, sample sizes, t-values, or *p*-values).	Studies that did not report key statistics and for which the necessary information could not be retrieved after contacting the corresponding author.
Publication language	English-language publications	Non-English publications

**Table 2:** Quality assessment through QualSyst.

No.	References	I	II	III	IV	V	VI	VII	VIII	IX	X	XI	XII	XIII	XIV	Rating
1	Nadarevic et al. (2020)	2	2	2	2	2	0	0	2	2	2	2	2	2	2	High quality
2	Gearhart et al. (2020)	2	2	2	2	2	0	0	2	2	2	2	2	2	2	High quality
3	Jahng and Littau (2015)	2	1	2	2	2	0	0	2	2	2	2	2	2	2	High quality
4	Westerman et al. (2014)	2	1	2	2	2	0	0	2	2	2	2	1	2	2	Moderate quality
5	Mena et al. (2020)	2	2	2	2	2	0	0	2	2	2	2	2	2	2	High quality
6	Overgaard (2021)	2	2	2	2	2	0	0	1	2	2	2	2	2	2	High quality
7	Lin et al. (2016)	2	2	2	2	2	0	0	2	2	2	2	1	2	2	High quality
8	Bucy (2003)	2	1	2	2	2	0	0	2	1	2	2	2	2	2	Moderate quality
9	Millet et al. (2024)	2	2	2	2	2	0	0	2	2	2	2	2	2	2	High quality
10	Malhotra and Shin (2025)	2	2	2	2	2	0	0	2	2	2	2	2	2	2	High quality
11	Borah and Xiao (2018)	2	2	2	2	2	0	0	2	2	2	2	2	2	2	High quality
12	De Vries (2019)	2	2	2	2	2	0	0	2	2	2	2	2	2	2	High quality
13	Gao et al. (2015)	2	2	2	2	2	0	0	2	1	2	2	2	2	2	High quality
14	Lin and Spence (2018)	2	2	2	2	2	0	0	2	2	2	2	1	2	2	High quality
15	Pjesivac et al. (2018)	2	2	2	2	2	0	0	2	2	2	2	2	2	2	High quality
16	Traberg et al. (2024)	2	2	2	2	2	0	0	2	2	2	2	2	2	2	High quality
17	Kuutila et al. (2024)	2	2	2	2	2	0	0	2	2	2	2	1	2	2	High quality
18	Liao et al. (2026)	2	2	2	2	2	0	0	2	2	2	2	2	2	2	High quality

I. Question/objective sufficiently described? II. Study design evident and appropriate to answer the study question? III. Method of subject/comparison group selection or source of information/input variables described and appropriate? IV. Subject (and comparison group, if applicable) characteristics sufficiently described? V. If random allocation to treatment group was possible, is it described? VI. If interventional and blinding of investigators was possible, was it reported? VII. If interventional and blinding of subjects was possible, was it reported? VIII. Outcome and (if applicable) exposure measure(s) well defined and robust to measurement/misclassification bias? Means of assessment reported? IX. Sample size appropriate? X. Analysis described and appropriate? XI. Some estimate of variance is reported for the main results? XII. Controlled for confounding? XIII. Results reported in sufficient detail? XIV. Conclusions supported by the results?

**Table 3:** Study characteristics of the included studies.

No.	References	Sample Size	Heuristic Cue Type	Participant Type	Perceived Credibility Measurement	Experiment Setting
1	Nadarevic et al. (2020)	175	Source Cue Type (Expert source)	Student	Unidimensional	Online
2	Gearhart et al. (2020)	447	Social cue type (Opinion Climate) (Congruent vs. Control)	Non-student	Unidimensional	Online
3	Jahng and Littau (2015)	154	Social cue type (Structural Cue (Interactivity))	Student	Multidimensional	Online
4	Westerman et al. (2014)	125	Social cue type (Temporal Cue (Update Recency))	Student	Multidimensional	Online
5	Mena et al. (2020)	478	Social cue type (Bandwagon Cue)	Non-student	Unidimensional	Online
6	Overgaard (2021)	540	Social cue type (Affective Cue)	Non-student	Multidimensional	Online
7	Lin et al. (2016)	249	Source Cue Type (Official source)	Student	Multidimensional	Offline
8	Bucy (2003)	168	Source Cue Type (Official source)	Student	Multidimensional	Offline
9	Millet et al. (2024)	542	Source Cue Type (Credible source)	Non-student	Unidimensional	Online
10	Malhotra and Shin (2025)	120	Social cue type (Visibility Cue)	Non-student	Unidimensional	Online
11	Borah and Xiao (2018)	340	Source Cue Type (Expert source)	Student	Unidimensional	Online
12	De Vries (2019)	300	Social cue type (Engagement Ratio Cue)	Non-student	Unidimensional	Online
13	Gao et al. (2015)	80	Source Cue Type (Credible source)	Student	Unidimensional	Offline
14	Lin and Spence (2018)	284	Source Cue Type (Expert source)	Student	Multidimensional	Offline
15	Pjesivac et al. (2018)	256	Social cue type (Affective Cue)	Student	Multidimensional	Online
16	Traberg et al. (2024)	5400	Source Cue Type (Expert source)	Non-student	Multidimensional	Online
17	Kuutila et al. (2024)	4187	Source Cue Type (Expert source)	Non-student	Unidimensional	Online
18	Liao et al. (2026)	162	Source Cue Type (Expert source)	Non-student	Unidimensional	Online

**Table 4:** Summary of overall mean effect size and test for heterogeneity.

Effect Size and 95% CI			Heterogeneity Test			Tau-Squared			Test of Null (Two-Tailed)	
k	g	95% CI	Q	*p*	I-Squared	Tau Squared	Standard Error	Tau	Z	*p*
19	0.307	[0.217, 0.396]	84.247	<0.001	78.634	0.025	0.018	0.158	6.691	<0.001

**Table 5:** Summary of Subgroup Analysis Results.

Moderator Variables	k	Hedges’ g	SE	95% CI	I-Squared	Q_W_	*p*
Heuristic Cue Type	Q_B_ = 7.815 (*p* = 0.005)						
Source cue	11	0.406	0.066	[0.276–0.536]	86.490	73.846	0.000
Social cue	8	0.185	0.043	[0.100–0.269]	8.475	7.657	0.364
Participant Type	Q_B_ = 4.367 (*p* = 0.037)						
Student	10	0.427	0.093	[0.245–0.610]	75.558	36.823	0.000
Non-student	9	0.210	0.046	[0.119–0.300]	73.906	30.658	0.000
Perceived credibility measurement	Q_B_ = 0.424 (*p* = 0.515)						
multidimensional measures	8	0.350	0.092	[0.169–0.530]	84.665	45.648	0.000
unidimensional measures	11	0.280	0.055	[0.172–0.388]	67.162	30.452	0.001
Experiment setting	Q_B_ = 2.980 (*p* = 0.084)						
Online	15	0.254	0.044	[0.168–0.340]	74.135	54.126	0.000
Offline	4	0.530	0.154	[0.228–0.833]	75.686	12.339	0.006

**Table 6:** Meta-Regression Results for Continuous Moderators.

Variable	Estimate	SE	95% CI	Z	*p*	QM	df
Sample size	−0.000	0.000	[−0.0001–−0.0000]	−0.86	0.3916	0.73	1
Publication year	−0.0208	0.0093	[−0.0390–−0.0026]	−2.24	0.0254	5.00	1

## Data Availability

The raw data supporting the conclusions of this article will be made available by the authors on request.

## Supplementary Materials

The following supporting information can be downloaded at: https://www.mdpi.com/article/10.3390/bs16071184/s1, Table S1: Complete Search Strategy.
