# Supplementary material for: How Heuristic Credibility Cues Shape Perceived Credibility on Social Media: A Meta-Analysis of Experimental Research
Source: Behav Sci (Basel). 2026 Jul 13;16(7):1184. doi: 10.3390/bs16071184 (PMC13404863; doi:10.3390/bs16071184)
Supplement: Supplementary file 1 [file behavsci-16-01184-s001.zip › behavsci-4385877-supplementary.pdf]

Supplementary Table of Complete Search Strategy

Database	Search Field	Complete Search String	Date Range	Additional Limits
Web of Science Core Collection	Abstract	AB=(("social media"OR "social networking site*"OR SNS) AND ("perceived credibility" OR credib* OR trust OR believab* OR trustworth*) AND ("heuristic cue*" OR heuristic OR cue* OR "source cue*" OR "social cue*" OR "endorsement cue*") AND (experiment* OR experimental OR RCT))	10 April 2026	Language: English; Document type: Article
Scopus	TITLE-ABS-KEY	(("social media" OR "social networking site*" OR sns) AND ("perceived credibility" OR credib* OR trust OR believab* OR trustworth*) AND ("heuristic cue*" OR heuristic OR cue* OR "source cue*" OR "social cue*" OR "endorsement cue*") AND (experiment* OR experimental OR rct))	10 April 2026	Language: English; Document type: Article
PubMed	Title/Abstract	((((((((((((((social media[Title/Abstract]) OR (social networking site[Title/Abstract])) OR (SNS[Title/Abstract])) AND (perceived credibility[Title/Abstract])) OR (credib*[Title/Abstract])) OR (trust[Title/Abstract])) OR (believab*[Title/Abstract])) OR (trustworth*[Title/Abstract])) AND (heuristic cue*[Title/Abstract])) OR (heuristic[Title/Abstract])) OR (source cue*[Title/Abstract])) OR (social cue*[Title/Abstract])) OR (endorsement cue*[Title/Abstract])) AND (experiment*[Title/Abstract])) OR (experimental[Title/Abstract])) OR (RCT[Title/Abstract])	10 April 2026	Free full text, English Abstract
PsycINFO	Abstract	AB（"social media" OR "social networking site*" OR SNS) AND ("perceived credibility" OR credib* OR trust OR believab* OR trustworth*) AND (heuristic OR "heuristic cue" OR "source cue" OR "social cue" OR "endorsement cue") AND (experiment* OR experimental OR RCT)	10 April 2026	Language: English; Peer-reviewed articles
Communication & Mass Media Complete	Abstract (AB)	AB(("social media" OR "social networking site*" OR SNS) AND ("perceived credibility" OR credib* OR trust OR believab* OR trustworth*) OR ("heuristic cue*" OR heuristic OR cue* OR "source cue*" OR "social cue*" OR "endorsement cue*") AND (experiment* OR experimental OR RCT))	10 April 2026	Language: English; Peer-reviewed articles
Google Scholar	Full text	"social media" credibility heuristic experiment	10 April 2026	Results sorted by relevance First 500 records screened Duplicates removed before full-text review
